# Fluoroquinolone-Free Prophylaxis and Impact on Bacterial Infections, Resistance, and Survival in Allo-HSCT: 5-Year Follow-up of the BATMO Protocol (Best Anti-microbical Therapy Trapianto di Midollo Osseo)

**DOI:** 10.1093/ofid/ofag511

**Published:** 2026-08-13

**Authors:** Daniele Avenoso, Mirko Farina, Simone Ferri, Lorenzo Masina, Liana Signorini, Silvia Lorenzotti, Evelyn Van Hauwermeiren, Biagio Francesco Canino, Silvia Corbellini, Francesca Caccuri, Gabriele Magliano, Enrico Morello, Vera Radici, Simona Bernardi, Federica Re, Alessandro Leoni, Arnaldo Caruso, Domenico Russo, Michele Malagola

**Affiliations:** Unit of Blood Diseases and Bone Marrow Transplantation, Department of Clinical and Experimental Science, University of Brescia, ASST Spedali Civili di Brescia, Brescia, Italy; Unit of Blood Diseases and Bone Marrow Transplantation, Department of Clinical and Experimental Science, University of Brescia, ASST Spedali Civili di Brescia, Brescia, Italy; Unit of Blood Diseases and Bone Marrow Transplantation, Department of Clinical and Experimental Science, University of Brescia, ASST Spedali Civili di Brescia, Brescia, Italy; Unit of Blood Diseases and Bone Marrow Transplantation, Department of Clinical and Experimental Science, University of Brescia, ASST Spedali Civili di Brescia, Brescia, Italy; Unit of Vaccinations and Infectious Diseases Surveillance, ASST Garda, 25015 Desenzano D/G, Brescia, Italy; Unit of Infectious and Tropical Diseases, ASST Spedali Civili di Brescia, Brescia, Italy; Unit of Infectious and Tropical Diseases, ASST Spedali Civili di Brescia, Brescia, Italy; Department of Molecular and Translational Medicine, Institute of Microbiology, University of Brescia, ASST Spedali Civili, Brescia, Italy; Department of Molecular and Translational Medicine, Institute of Microbiology, University of Brescia, ASST Spedali Civili, Brescia, Italy; Department of Molecular and Translational Medicine, Institute of Microbiology, University of Brescia, ASST Spedali Civili, Brescia, Italy; Unit of Blood Diseases and Bone Marrow Transplantation, Department of Clinical and Experimental Science, University of Brescia, ASST Spedali Civili di Brescia, Brescia, Italy; Unit of Blood Diseases and Bone Marrow Transplantation, Department of Clinical and Experimental Science, University of Brescia, ASST Spedali Civili di Brescia, Brescia, Italy; Unit of Blood Diseases and Bone Marrow Transplantation, Department of Clinical and Experimental Science, University of Brescia, ASST Spedali Civili di Brescia, Brescia, Italy; Unit of Blood Diseases and Bone Marrow Transplantation, Department of Clinical and Experimental Science, University of Brescia, ASST Spedali Civili di Brescia, Brescia, Italy; Centro di Ricerca Emato-Oncologica AIL (CREA), ASST Spedali Civili di Brescia, Brescia, Italy; Unit of Blood Diseases and Bone Marrow Transplantation, Department of Clinical and Experimental Science, University of Brescia, ASST Spedali Civili di Brescia, Brescia, Italy; Centro di Ricerca Emato-Oncologica AIL (CREA), ASST Spedali Civili di Brescia, Brescia, Italy; Unit of Blood Diseases and Bone Marrow Transplantation, Department of Clinical and Experimental Science, University of Brescia, ASST Spedali Civili di Brescia, Brescia, Italy; Centro di Ricerca Emato-Oncologica AIL (CREA), ASST Spedali Civili di Brescia, Brescia, Italy; Department of Molecular and Translational Medicine, Institute of Microbiology, University of Brescia, ASST Spedali Civili, Brescia, Italy; Unit of Blood Diseases and Bone Marrow Transplantation, Department of Clinical and Experimental Science, University of Brescia, ASST Spedali Civili di Brescia, Brescia, Italy; Unit of Blood Diseases and Bone Marrow Transplantation, Department of Clinical and Experimental Science, University of Brescia, ASST Spedali Civili di Brescia, Brescia, Italy

**Keywords:** allo-HSCT, antibiotic stewardship, BATMO, fluorochinolone, neutropenic fever

## Abstract

**Background:**

Infectious complications and antimicrobial resistance (AMR) are leading causes of morbidity and non-relapse mortality (NRM) in patients undergoing allogeneic hematopoietic stem cell transplantation (allo-HSCT). The BATMO protocol, implemented in 2019, removed fluoroquinolone prophylaxis and introduced a personalized, stewardship-based antimicrobial approach.

**Methods:**

We retrospectively analyzed 323 allo-HSCT recipients transplanted between 2016 and 2023 at a single center, comparing outcomes in patients before (n = 115) and after (n = 208) BATMO implementation. Primary endpoints included overall survival (OS), NRM, incidence and microbiology of bloodstream infections (BSIs), antimicrobial resistance trends, and carbapenem use. Kaplan-Meier and cumulative incidence methods were employed.

**Results:**

Despite a significant increase in 30-day Gram-negative BSI incidence post-BATMO (24% vs 15%, *P* = .03), OS and NRM were similar between the two cohorts. Notably, the post-BATMO cohort included a substantially higher proportion of high-risk transplants, with a marked increase in haploidentical procedures and broader use of post-transplant cyclophosphamide. Interestingly, fluoroquinolone-resistant *E. coli* strains declined from 100% to 46% (*P* = .003), and overall Gram-negative resistance dropped from 75% to 36% (*P* = .006). Use of carbapenems remained unchanged (43% vs 53%, *P* = .1).

**Conclusions:**

The BATMO protocol demonstrates that withdrawal of fluoroquinolone prophylaxis and implementation of a targeted antimicrobial strategy can reduce resistance without compromising transplant outcomes. These findings support the long-term safety and efficacy of stewardship-based approaches in allo-HSCT.

Key Findings of the BATMO Study:

- Withdrawal of fluoroquinolone prophylaxis within the BATMO protocol was associated with a higher incidence of early Gram-negative bloodstream infections within 30 days after allo-HSCT.
- Despite the increase in early BSI incidence, overall survival and non-relapse mortality remained comparable between the pre- and post-BATMO eras.
- The post-BATMO cohort included a higher proportion of high-risk transplants, including increased use of haploidentical donors and post-transplant cyclophosphamide.
- Fluoroquinolone resistance among Gram-negative isolates significantly decreased after BATMO implementation, with E. coli resistance declining from 100% to 46%.
- Carbapenem use did not increase following the implementation of the stewardship-based BATMO protocol.

Infectious complications represent a major cause of morbidity and non-relapse mortality (NRM) in patients undergoing allogeneic hematopoietic stem cell transplantation (allo-HSCT) [1, 2]. These complications are particularly challenging due to the profound and prolonged immunosuppression associated with conditioning regimens, graft-versus-host disease (GvHD), and the use of immunosuppressive therapies [3]. The risk of infection is further amplified by the increasing global burden of antimicrobial resistance (AMR), which compromises the efficacy of standard prophylactic and empirical treatments [4].

Historically, fluoroquinolone prophylaxis has been widely adopted to reduce the incidence of Gram-negative bloodstream infections (BSI) in neutropenic patients [5–9]. However, the widespread use of fluoroquinolones has been associated with the emergence of resistant strains, including multidrug-resistant (MDR) and extensively drug-resistant (XDR) organisms, particularly *Escherichia coli* and *Klebsiella pneumoniae* [10–14]. These pathogens have been linked to increased infection-related mortality, prolonged hospitalization, and limited therapeutic options in the allo-HSCT setting [12–14].

In response to these concerns, a novel antimicrobial stewardship strategy known as the BATMO (“*Best Antimicrobial Therapy in Trapianto di Midollo Osseo*” – “*Best Antimicrobial Therapy in Bone Marrow Transplant*”) protocol was implemented in 2019 at the Adult Bone Marrow Transplant Unit of the Spedali Civili of Brescia, Italy. This protocol introduced key modifications, including the discontinuation of fluoroquinolone prophylaxis, targeted use of letermovir for CMV prophylaxis in CMV-positive recipients, anti-fungal prophylaxis with posaconazole in high-risk patients (eg, haploidentical transplants), and personalized antimicrobial treatment based on colonization status and local epidemiology [15, 16]. The overarching goal was to reduce the selection pressure driving AMR, while maintaining effective infection control and safeguarding patient outcomes.

The initial results from a two-year follow-up regarding the absence of fluoroquinolone prophylaxis in neutropenic patients undergoing HSCT showed promising signs of efficacy and safety, with stable overall survival (OS) and NRM despite a modest increase in early BSI [16]. However, long-term data are essential to fully assess the durability of these findings and the broader impact on resistance profiles and infectious outcomes.

This study presents the 5-year follow-up of the BATMO protocol, evaluating its impact on infectious complications, antimicrobial resistance trends, and patient survival in a large cohort of allo-HSCT recipients. Our findings aim to inform clinical practice and contribute to the ongoing debate on the optimization of antimicrobial strategies in immunocompromised patients.

## METHODS

### Best Antimicrobical Therapy in Trapianto di Midollo Osseo (TMO)—BATMO

The BATMO program was designed in collaboration with our colleagues from the Infectious Diseases and Microbiology units, according to revised data on local epidemiology and the outcome of patients submitted to allo-HSCT from 2016 to 2018. Compared to the historical approach, the main features of this program concern both the prophylaxis and the therapeutic management of infectious complications. Firstly, antibacterial prophylaxis with levofloxacin, during the phase of neutropenia, was suspended. Secondly, antifungal prophylaxis with fluconazole was reserved only for patients at low risk of invasive fungal disease (IFD) according to the score proposed by Stanzani and Colleagues [17]. Conversely, in high-risk patients, posaconazole was introduced. Moreover, for all patients, fungal prophylaxis was supplemented with aerosolized liposomal amphotericin B according to Rijnders et al. (2.5 ml/daily of a 5 mg/ml solution in the first week, followed by 2.5 ml for 2 consecutive days every week until engraftment or a need for antifungal treatment) [18]. Thirdly, all CMV IgG-positive patients received letermovir prophylaxis from day 0 to day +100 [19]. Acyclovir from the day of SCT and co-trimoxazole from the day of engraftment remained the standard prophylaxis for herpes simplex and *Pneumocystis jirovecii*, until immunological reconstitution (CD4+ cells greater than 250/mmc). The monitoring of viral reactivations consisted of CMV, HHV6, and EBV DNAemia assessment weekly from the start of conditioning up to day +90, and then every 2 weeks until day +180. For other viruses (eg, respiratory viruses including SARS CoV2) the monitoring was performed based on symptoms (eg, cough, fever, cold). Toxoplasma monitoring with molecular biology was performed only in the case of symptoms potentially related to this infection. Focusing on fungal infections, the galactomannan antigen was monitored in peripheral blood weekly, from the day of SCT until day +90 and then every 2 weeks until day +180. Although serum galactomannan has reduced sensitivity during mold-active prophylaxis, it was retained as part of our surveillance strategy for breakthrough invasive fungal infections and was interpreted together with clinical findings and therapeutic drug monitoring of posaconazole. In high-risk patients, such as those developing acute GvHD requiring systemic corticosteroids, viral and fungal monitoring was continued on a weekly or twice a week according to corticosteroid dose, irrespective of the time from transplantation, rather than being limited to the routine post-transplant schedule. A core principle of the BATMO program is the rational and selective use of antibiotics, with the overarching goal of limiting unnecessary exposure and thereby mitigating the risk of emerging antimicrobial resistance. Moreover, intensive surveillance for bacterial colonization was performed by obtaining rectal, pharyngeal, nasal, and skin swabs at hospital admission and then weekly throughout the neutropenic phase. These surveillance cultures were used to detect colonization with multidrug-resistant organisms according to institutional infection-control procedures. Colonization results did not prompt antimicrobial treatment in asymptomatic patients but were incorporated into empirical antibiotic selection in the event of febrile neutropenia. In particular, colonization with carbapenemase-producing *K. pneumoniae* guided the use of ceftazidime-avibactam or ceftolozane-tazobactam as first-line empirical therapy, whereas carbapenems were otherwise reserved for second-line treatment or microbiologically documented indications. First-line antibiotics in the case of fever during neutropenia consisted of piperacillin-tazobactam or ceftazidime or cefepime together with glycopeptide if Gram-positive infection was suspected. In addition to the discontinuation of fluoroquinolone prophylaxis, the BATMO protocol promotes a more judicious and selective use of carbapenems. These agents are reserved for second-line treatment in cases of first-line failure or are employed as first-line therapy only when guided by antimicrobial susceptibility testing. Furthermore, novel antibiotics such as ceftolozane-tazobactam and ceftazidime-avibactam are used as first-line options exclusively in patients colonized with *K. pneumoniae* carbapenemase (KPC)-producing *K. pneumoniae* [20, 21]

Antibacterial prophylaxis in the pre-BATMO era consisted of levofloxacin 500 mg once daily administered from the onset of neutropenia until neutrophil engraftment or the initiation of empiric broad-spectrum antibiotics in case of fever.

### Study Design and Rationale

This is a retrospective, single-center, observational cohort study using a historical comparison between two consecutive transplant eras (pre- and post-BATMO implementation). The primary objective was to assess associations between the implementation of the BATMO protocol and infectious complications, antimicrobial resistance patterns, and transplant outcomes. Given the non-randomized design, the study was not intended to establish causal inference but rather to provide associational and epidemiologic insights.

For the purposes of this study, BSI were analyzed only if occurring within the first 30 days after transplantation (early BSI). The primary outcomes of the study were the incidence of Gram-negative BSI within 30 days after transplantation, overall survival, and non-relapse mortality. This time window was selected because it encompasses the period of highest infection risk, characterized by chemotherapy-induced mucosal injury, profound neutropenia, and intensive immunosuppressive therapy. Beyond day +30, time-dependent factors such as changes in immunosuppressive therapy, GVHD treatment, and disease relapse increasingly influence infection risk. Patients were classified into two exposure groups according to transplant era: a pre-BATMO cohort including patients transplanted between 2016 and 2018 and a post-BATMO cohort including patients transplanted after implementation of the BATMO protocol between 2019 and 2023. Written informed consent for data collection and analysis was obtained from all patients. The study was approved by the local ethics committee and conducted in accordance with the Declaration of Helsinki.

### Statistical Analysis

All data were collected from the Adult Bone Marrow Transplantation Unit and the Department of Microbiology at the Spedali Civili of Brescia. Continuous variables were described as median and range; categorical variables, as frequencies. Overall survival (OS) was estimated using Kaplan-Meier curves; OS was defined as time from HSCT to death from any cause or the last follow-up for living patients. A Fine–Gray regression model for competing risks was utilized for cumulative incidence analysis of non-relapse mortality (NRM), relapse (CIR), and acute and chronic GVHD. NRM was defined as death from any cause other than progression of the underlying malignancy, with death due to relapse as a competing event. Relapse was defined as recurrence of the underlying hematologic malignancy; death due to any other cause was a competing event for this analysis. For cumulative incidence analysis of GVHD, death without aGVHD in the first 100 days was considered a competing event for the aGVHD endpoint, whereas relapse or death in the absence of cGVHD was considered a competing event for the cGVHD outcome. To account for potential chronologic bias and secular changes in transplant practice, additional analyses were performed on the entire cohort. Overall survival was evaluated using Cox proportional hazards models, with the BATMO period (pre- vs post-BATMO) as the exposure variable. The models were adjusted for age at transplant, disease category, HCT-CI, stem cell source (BM vs PBSC), donor type (matched related, matched unrelated, haploidentical), and GvHD prophylaxis (including PTCy use). Variables included in the multivariable models were selected based on univariable analyses and clinical relevance. Age and HCT-CI were retained irrespective of their univariable significance because of their established prognostic importance in the allogeneic HSCT setting. Non-relapse mortality was assessed using Fine–Gray subdistribution hazards models, with the BATMO period (pre- vs post-BATMO) as the exposure variable and adjustment for the same covariates. Proportional hazards and proportional subdistribution hazards assumptions were assessed using Schoenfeld residuals and time-interaction terms as appropriate. A sensitivity analysis restricted to PBSC recipients was conducted to reduce heterogeneity related to stem cell source and to partially mitigate secular practice changes. Median follow-up was estimated using the reverse Kaplan–Meier method. Categorical variables were compared using Fisher's exact test when expected cell counts were small. Statistical analyses were performed using RStudio software (version 4.3.2).

## RESULTS

### Patients’ Characteristics

A total of 323 patients who underwent allo-HSCT at the Adult Bone Marrow Transplantation Unit of the Spedali Civili of Brescia between 2016 and 2023 were included in the analysis (Table 1). Of these, 115 patients belonged to the pre-BATMO cohort (transplanted between 2016 and 2018), while 208 patients were included in the post-BATMO cohort (transplanted between 2019 and 2023, after implementation of the BATMO protocol).

**Table 1.** Clinical and Transplant Characteristics of the Study Population

Characteristic	Pre-BATMO	Post-BATMO
Sex		
Male	68 (60%)	138 (66%)
Female	47 (45.21%)	70 (34%)
Age at transplant (y)	Median: 54 (IQR: 43–61)	Median: 54 (IQR: 43–61)
Disease		
Acute leukemia and MDS	83 (72%)	126 (61%)
Lymphoma	9 (8%)	32 (15%)
Multiple myeloma	5 (4%)	12 (6%)
Other	18 (15%)	38 (21.63%)
Pre-transplant therapy lines		
Frontline only	45 (40%)	86 (41%)
≥2 lines	70 (60%)	122 (59%)
Conditioning regimen		
Myeloablative	55 (48.69%)	118 (53%)
Reduced intensity	60 (51.21%)	98 (47%)
Stem cell source		
Bone marrow	32 (28%)	16 (7%)
Peripheral blood	82 (71%)	192 (93%)
Cord blood	1 (1%)	0 (0.00%)
Donor type		
HLA-matched related donor	38 (33%)	46 (22%)
HLA-matched unrelated donor	56 (49%)	98 (47%)
Haploidentical donor	21 (18%)	64 (31%)
GvHD prophylaxis		
CsA + MMF or MTX	10 (9%)	4 (2%)
CsA ± MTX or MTX + ATG/ATLG	91 (79%)	160 (70%)
Post-transplant cyclophosphamide	6 (5%)	44 (21%)
Other	8 (7%)	2 (1%)

Abbreviations: CsA, ciclosporin A; MMF, mycophenolate mofetil; MTX, methotrexate; GvHD, graft-versus-host disease.

Overall, the clinical characteristics of the two cohorts were comparable, with the exception of a higher prevalence of acute leukemias in the pre-BATMO era. No significant differences were observed between the two groups in terms of conditioning intensity. However, transplant practices evolved over time. In the post-BATMO cohort, the use of bone marrow as a stem cell source significantly decreased (28% vs 7%; *P* < .001), with a corresponding increase in peripheral blood stem cell transplantation (71% vs 92%; *P* < .001).

Differences were also observed in donor type. The proportion of HLA-matched related donors decreased from 33% to 22% (*P* = .034), whereas haploidentical transplants increased from 18% to 31% in the post-BATMO period (*P* = .011). In terms of graft-versus-host disease (GvHD) prophylaxis, the use of post-transplant cyclophosphamide increased substantially in the post-BATMO cohort (5.2% vs 21%; *P* < .001), while the use of cyclosporin combined with mycophenolate mofetil or methotrexate progressively decreased (9% vs 2%; *P* = .007).

### Early Microbiologically Documented Infections and Bloodstream Infections

Table 2 summarizes the incidence of microbiologically documented infections (Table 2) and BSI occurring within the first 30 days after transplantation (Table 3). Overall, microbiologically documented infections within the first 30 days after transplantation were significantly more frequent in the post-BATMO cohort than in the pre-BATMO cohort (69% vs 49%; *P* = .0008). This increase was mainly driven by a higher incidence of Gram-negative infections (30% [62/208] vs 18% [21/115]; *P* = .03), whereas the incidence of Gram-positive and fungal infections remained similar between the two eras.

**Table 2.** Microbiological Documented Infections Within the First 30 Days After SCT in the Pre- and Post-BATMO Era

Variable	Total	Pre-BATMO	Post-BATMO	*P V*alue
Episodes/Pts (%)	200/323 (62)	57/115 (49)	143/208 (69)	.**0008**
Gram positive	106/323 (33)	35/115 (30)	71/208 (34)	.46
Gram negative	83/323 (26)	21/115 (18)	62/208 (30)	.**03**
Polymicrobial infections	19/323 (2%)	3/115 (3)	16/208 (8)	.93
Fungi	3/323 (1)	1/115 (1)	2/208 (1)	>.99

Bold values indicate statistical significance (*P* < .05).

**Table 3.** Patients With at Least One BSI Within the First 30 Days After SCT in the Pre- and Post-BATMO Era

Variable	Total (Pts - %)	Pre-BATMO (Pts - %)	Post-BATMO (Pts - %)	*P V*alue
Total	162/323 (50)	46/115 (40)	116/208 (56)	.**008**
Gram positive	78/323 (24)	26/115 (23)	52/208 (25)	.68
Gram negative	68/323 (21)	16/115 (14)	50/208 (24)	.**03**
Polymicrobial infections	19/323 (6)	3/115 (3)	16/208 (8)	.08

Bold values indicate statistical significance (*P* < .05).

Similarly, the proportion of patients developing Gram-negative BSI within 30 days after transplantation increased from 14% (16/115) in the pre-BATMO era to 24% (50/208) after BATMO implementation (*P* = .03).

The distribution of isolated pathogens is reported in Supplementary Figure 1. Among Gram-positive organisms, coagulase-negative staphylococci represented the most frequently isolated pathogens in both periods (54% vs 45% in the pre- and post-BATMO eras, respectively). Other Gram-positive species showed similar distributions, with the exception of Enterococcus faecium, which increased from 3% to 14% in the post-BATMO era.

Among Gram-negative pathogens, *E. coli* remained the most frequently isolated organism in both cohorts (52% vs 42% in the pre- and post-BATMO eras, respectively). An increase in *K. pneumoniae* detection was observed in the post-BATMO period (14% vs 34%), consistent with broader epidemiological trends reported in hospitalized and hematological patient populations.

The prevalence of rectal swab colonization at admission was similar between the two cohorts (10% in the pre-BATMO vs 16% in the post-BATMO era; *P* = .11). In both groups, extended-spectrum beta-lactamase–producing E. coli was the most frequent isolate. In the post-BATMO cohort, a higher number of vancomycin-resistant enterococci was detected (11 cases vs none in the pre-BATMO cohort).

### Transplant Outcomes

**Figure 1.**
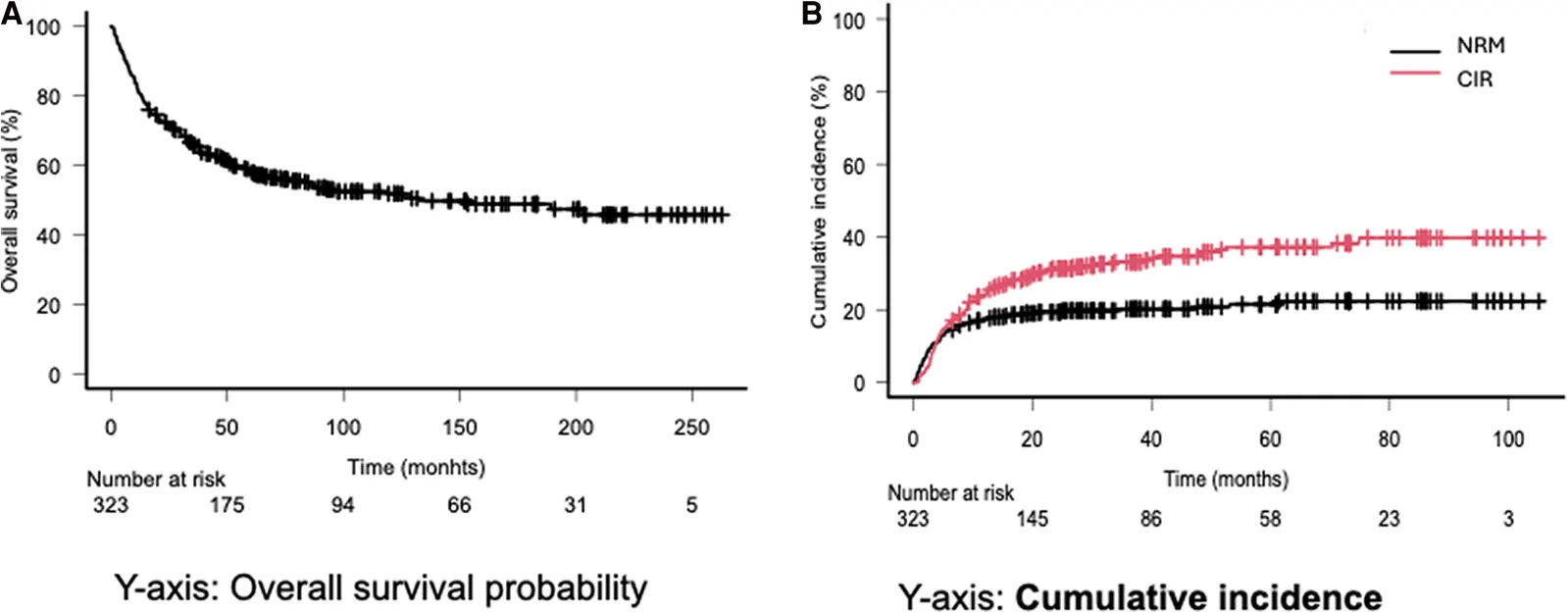
OS (*A*), NRM, and CIR (*B*) of the 323 patients of the study (Median OS 132.8 m; 2-year NRM and CIR: 19.4% and 31.4%).

**Figure 2.**
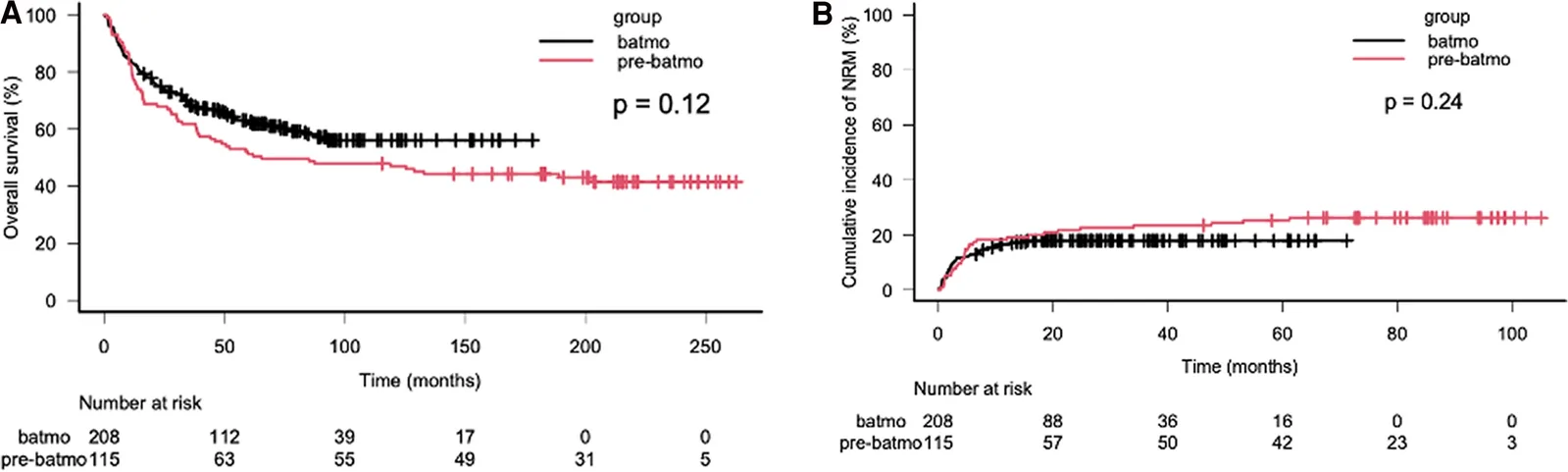
OS (*A*) and NRM (*B*) according to the *eras* (pre vs post-BATMO) (Median OS 65.3 m vs not reached; *P* = .12. 2-year NRM 22% vs 18%; *P* = .24).

Long-term transplant outcomes were evaluated in the entire cohort. No significant differences were observed between the two eras in terms of time to neutrophil or platelet engraftment (data not shown). Overall survival (OS), non-relapse mortality (NRM), and cumulative incidence of relapse (CIR) are shown in Figure 1. The median OS for the entire cohort was 132.8 months (95% CI, 77.3–not reached). At 24 months, NRM was 19.4% (95% CI, 15.2–23.9), while the CIR was 31.4% (95% CI, 26.3–36.5). When outcomes were analyzed according to transplant era, median OS was not reached in the post-BATMO cohort (95% CI, 66–not reached) and was 65.3 months (95% CI, 38.4–202.9) in the pre-BATMO cohort (*P* = .12) (Figure 2*A*). Similarly, the 24-month NRM was 18% (95% CI, 13–23.5) in the BATMO era and 22% (95% CI, 14.7–29.7) in the pre-BATMO era (*P* = .24) (Figure 2*B*). In multivariable Cox regression analysis, BATMO period was not associated with higher mortality even after adjustment for age, HCT-CI, disease category, stem cell source, donor type, and GvHD prophylaxis (including PTCy). Only raised HCT-CI was associated with inferior OS. No significant associations were observed for age, stem cell source, donor type, or disease category (Supplementary Table 1). In competing-risk analysis using a Fine–Gray model, HCT-CI was independently associated with higher NRM (sHR 1.22; 95% CI, 1.08–1.38; *P* = .0017), whereas BATMO period and PTCy use were not significantly associated with NRM (Supplementary Table 2).

### Impact of Early Gram-negative BSI on Transplant Outcomes

**Figure 3.**
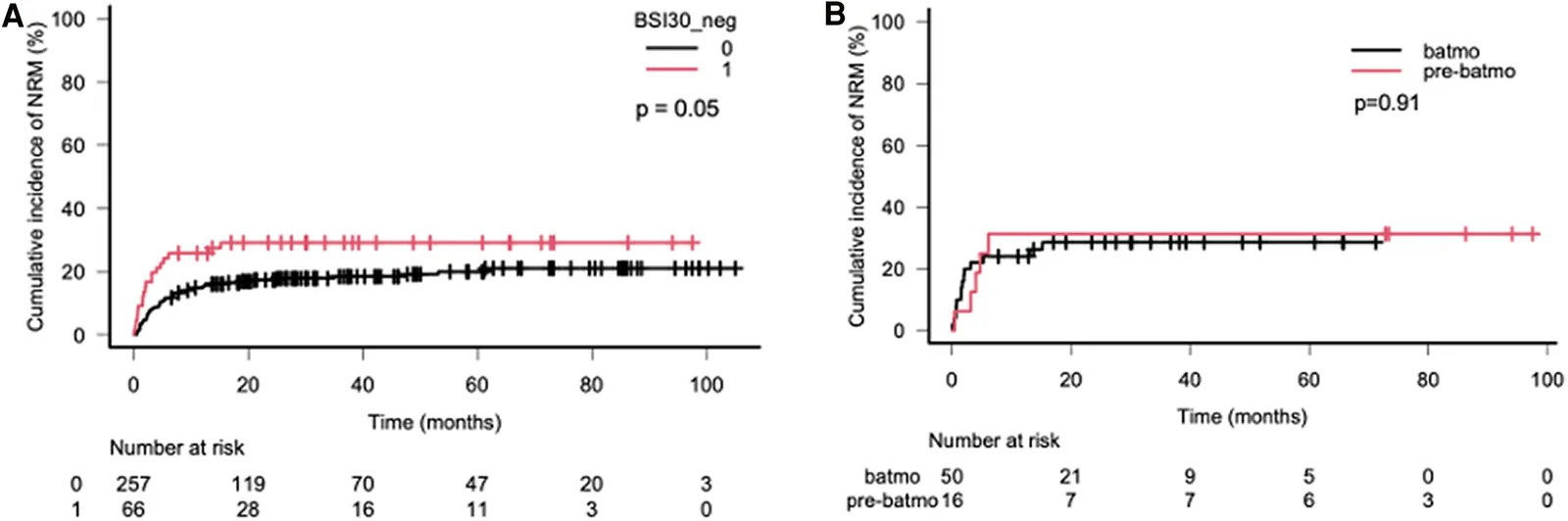
NRM of the patients according to the development of Gram-negative BSI by day +30 (yes vs no; 29.1% vs 17.3%; *P* = .05; *A*) and according to early Gram-negative BSI and protocol *era*s (pre vs post-BATMO; 31.2% vs 28.5%; *P* = .91; *B*).

**Figure 4.**
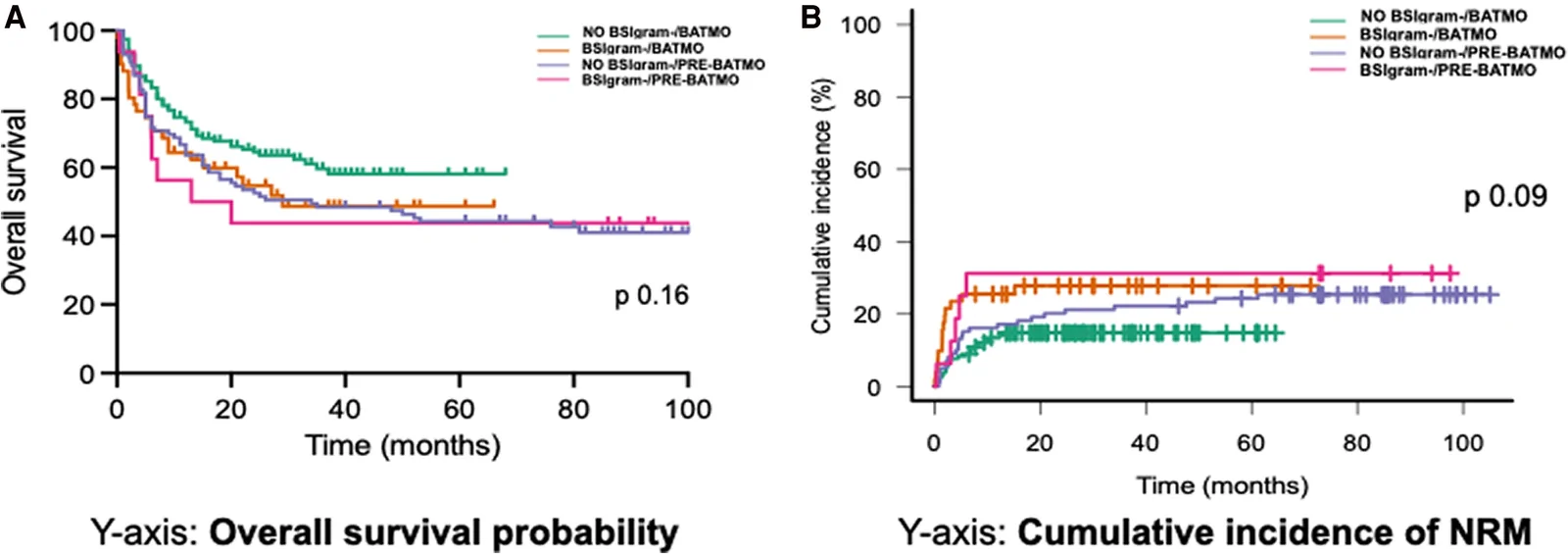
OS (*A*) and NRM (*B*) by combining the development of Gram-negative BSI by day +30 after SCT (yes vs no) with the *eras* (pre- and post-BATMO); (2-year OS 43.8% vs 52.5% and 55.6% vs 64.8%; *P* = .98 and *P* = .19, respectively; *A*); (2-year NRM 31.2% vs 20.2% and 26.4% vs 15.3%; *P* = .55 and *P* = .06, respectively; *B*).

When the entire cohort was analyzed according to the occurrence of Gram-negative BSI within 30 days after transplantation, patients who developed early Gram-negative BSI had higher NRM compared with those without infection (29.1% [95% CI, 18.6–40.5] vs 17.3% [95% CI, 12.9–22.2]; *P* = .05) (Figure 3*A*). However, when stratified by transplant era, the NRM among patients with early Gram-negative BSI was similar between the pre- and post-BATMO periods (31.2% vs 28.5% at 24 months; *P* = .91) (Figure 3*B*). Figure 4 shows OS and NRM according to the combined effect of transplant era and early Gram-negative BSI. In both eras, the development of early Gram-negative BSI was not associated with significantly different overall survival. In the pre-BATMO cohort, the 2-year OS was 43.8% in patients with BSI versus 52.5% in those without BSI (*P* = .89). In the post-BATMO cohort, the corresponding values were 55.6% and 64.8%, respectively (*P* = .19) (Figure 4*A*).

Similarly, NRM was comparable across transplant eras. However, the occurrence of early Gram-negative BSI was associated with a worse NRM, irrespective of the transplant period.

(26.4% vs 15.3%; *P* = .09) (Figure 4*B*).

Within the post-BATMO cohort, causes of death, causes of NRM, and the incidence of acute and chronic GvHD were not significantly different between patients with or without early Gram-negative BSI. However, a trend toward higher mortality from early infections (within 30 days after transplantation) was observed among patients with Gram-negative BSI (10% vs 3%; *P* = .06).

### Factors Associated With NRM in the BATMO Era

To identify factors associated with NRM in the post-BATMO cohort, univariable and multivariable analyses were performed (Table 4).

**Table 4.** Univariable and Multivariable Analysis of Factors Associated With NRM in the BATMO Era

	Univariable Analysis		Multivariable Analysis	
Variable	HR	95% CI	HR	95% CI
aGVHD	0.73	0.38–1.42	-	-
Grade 3–4 aGVHD	0.73	0.37–2.18	-	-
Age at transplant	1.00	0.98–1.03	-	-
Day 30 Gram-neg BSI	1.93	0.97–3.81	1.67	0.84–3.31
MAC conditioning	1.15	0.61–2.19	-	-
cGVHD	0.226	0.08–0.82	0.28	0.09–0.90
Haploidential donor	2.01	1.06–3.18	1.96	1.03–3.72
Donor sex	0.86	0.44–1.68	-	-
1st CR at SCT	0.69	0.34–1.7	-	-
HCT-CI ≥ 3	1.99	1.01–3.97	1.87	0.94–3.71
Patients’ sex (male)	1.39	0.72–2.68	-	-

In univariable analysis, chronic GvHD was associated with reduced NRM (HR 0.29; 95% CI, 0.08–0.82; *P* = .02). In contrast, haploidentical donor transplantation (HR 2.01; 95% CI, 1.06–3.81; *P* = .03) and HCT-CI ≥3 (HR 1.99; 95% CI, 1.01–3.97; *P* = .05) were associated with increased NRM.

In multivariable analysis, chronic GvHD remained independently associated with lower NRM (HR 0.28; 95% CI, .09–.90; *P* = .03), whereas haploidentical donor transplantation remained independently associated with higher NRM (HR 1.96; 95% CI, 1.03–3.72; *P* = .04).

### Antibiotic Resistance and Carbapenem Use

An additional objective of the study was to evaluate the impact of the BATMO antimicrobial stewardship strategy on antibiotic resistance among Gram-negative bacteria isolated from BSIs occurring within 30 days after transplantation.

No significant differences were observed between the two eras in the incidence of methicillin-resistant Staphylococcus aureus, methicillin-resistant Staphylococcus epidermidis, or vancomycin-resistant enterococci.

In contrast, a significant reduction in fluoroquinolone resistance was observed among Gram-negative isolates (Table 5). Fluoroquinolone-resistant *E. coli* strains decreased from 100% in the pre-BATMO cohort to 46% after BATMO implementation (*P* = .003). A similar trend was observed for *K. pneumoniae*, whereas resistance among Pseudomonas aeruginosa increased without reaching statistical significance. Overall, fluoroquinolone resistance among Gram-negative isolates decreased from 75% to 36% (*P* = .006).

**Table 5.** Antibiotic Resistance in Bacteria Isolated From BSIs at 30 Days

Bacteria Species	Pre-BATMO (n = 16)	Post-BATMO (n = 55)	*P V*alue
…	N (%)	N (%)	…
*E. coli*	**10**	**28**	…
ESBL+	1 (10)	3 (11)	1
CRE	0	…	1
Fluoroquinolone R	10 (90)	13 (46)	.003
MDRO	0	0	1
*K. pneumoniae*	**3**	**19**	…
ESBL+	1 (33)	5 (26)	1
CRE	1 (33)	2 (11)	.371
Fluoroquinolone R	2 (67)	4 (21)	.169
MDRO	1 (33)	2 (11)	.371
and XDRO	1 (33)	2 (11)	0371
*P. aeruginosa*	**3**	**8**	…
Fluoroquinolone R	0	3 (38)	.491
MDRO	0	2 (25)	1
and XDRO	0	2 (25)	1
Total	**16**	**55**	**.006**
ESBL+	2 (13)	8 (15)	1
CRE	1 (6)	2 (4)	.541
Fluoroquinolone R	12 (75)	20 (36)	.006
MDRO	1 (6)	4 (7)	1
and XDRO	1 (6)	4 (7)	1

Bold values indicate statistical significance (*P* < .05).

All multidrug-resistant organisms identified during the study fulfilled criteria for extensively drug-resistant organisms, with one such isolate observed in the pre-BATMO cohort and four in the post-BATMO cohort.

Finally, the proportion of patients receiving carbapenem therapy within 30 days after transplantation did not differ significantly between the two cohorts (43% vs 53%; *P* = .1), suggesting that the increase in early BSI incidence did not translate into greater carbapenem use.

## DISCUSSION

Five years after its implementation, the BATMO protocol has proven to be a safe, effective, and sustainable strategy for the management of infectious complications in patients undergoing allo-HSCT. This long-term follow-up analysis confirms and expands upon the encouraging results observed in the 2-year interim evaluation [16], providing robust evidence that antimicrobial stewardship, when implemented through a structured and personalized approach, does not compromise clinical outcomes—even in one of the most immunocompromised patient populations. Despite the deliberate withdrawal of fluoroquinolone prophylaxis—a measure historically associated with an increase in Gram-negative BSI rate and infection-related mortality [6–9]—OS and NRM remained stable across cohorts (Figure 2). Recent studies have explored the clinical consequences of discontinuing fluoroquinolone prophylaxis in hematopoietic stem cell transplantation. Stern et al. and Clerici et al. reported that omission of fluoroquinolone prophylaxis was associated with a higher incidence of early Gram-negative BSI but with lower antimicrobial resistance rates among Gram-negative pathogens. Importantly, these studies did not observe a significant increase in early infection-related mortality. Similarly, Guimarães et al. reported that withdrawal of fluoroquinolone prophylaxis was associated with reduced resistance among causative pathogens, despite a higher incidence of BSI. Our findings are consistent with these observations and further extend them by providing longer-term follow-up, showing that the increase in early BSI did not translate into worse overall survival or non-relapse mortality.

In 2021, the American Society of Transplantation and Cellular Therapies published the update of *Enterobacterales* management in patients submitted to allo-HSCT and confirmed the positive effect of fluoroquinolone prophylaxis on the incidence of Gram-negative infections but clearly highlighted the impact of extensive use of this prophylaxis and resistance both to fluoroquinolone and to other antibiotics (eg, beta-lactam antibiotics) [6]. According to our results, while the incidence of BSI within 30 days post-transplant did increase significantly in the post-BATMO cohort (Table 3), this rise was not associated with worsened long-term outcomes (Figure 4). Similarly, the overall incidence of microbiologically documented infections within the first 30 days after transplantation was also significantly higher in the post-BATMO cohort. This finding is consistent with the expected consequences of fluoroquinolone withdrawal and was primarily driven by Gram-negative bacterial infections. Importantly, despite the higher burden of documented infectious episodes, overall survival and non-relapse mortality remained unchanged, suggesting that the BATMO stewardship strategy enabled effective management of these infections without adversely affecting long-term transplant outcomes. In fact, survival analyses demonstrated comparable OS and NRM between the pre- and post-BATMO *eras*, suggesting that the absence of antibacterial prophylaxis did not translate to impaired outcomes. This stability in outcomes is particularly noteworthy considering the marked shift toward higher-risk transplants in the BATMO era. The increased proportion of haploidentical donors—procedures typically associated with higher infectious and immunologic complications—and the broader use of PtCY as GvHD prophylaxis would have been expected to negatively impact NRM. The absence of such deterioration reinforces the robustness of the BATMO strategy. The historical nature of the comparison represents an inherent limitation of the study. Secular changes in transplant practice, including increased use of PBSC, higher rates of haploidentical transplantation, and broader adoption of PTCy, may introduce residual confounding. Although we adjusted for these variables in multivariable models and performed sensitivity analyses restricted to more homogeneous subgroups, unmeasured confounding cannot be excluded. Therefore, our findings should be interpreted as associational rather than causal; also, the multivariable modeling confirmed that the stability of OS across eras was not explained by chronologic practice changes, supporting the robustness of our findings. In both cohorts, the occurrence of an early Gram-negative BSI is probably associated with an impaired outcome. In particular, in the BATMO *era* the incidence of all-cause mortality for these patients was 46% versus 39% for those without early Gram-negative BSI (*P* = .41; data not shown) and the 2-year NRM was 26% versus 15% NRM was 26% versus 15% (*P* = .096; Figure 4*B*). Deaths directly attributable to infection were twice as frequent in patients with early BSI, and infection-related mortality occurring within the first 30 days post-transplant reached 10% compared with 3% among patients without Gram-negative bacteremia.

Nevertheless, the multivariable analysis refined these findings, showing that although early Gram-negative BSI showed a trend toward higher NRM (HR 1.67; *P* = .14), it did not retain independent statistical significance after adjustment for other covariates. Instead, the use of a haploidentical donor (HR 1.96; *P* = .04) and a high HCT-CI score (≥3; HR 1.87; *P* = .08) emerged as the strongest predictors of increased NRM. In contrast, chronic GVHD showed a protective association (HR 0.28, 95% CI .09–.90, *P* = .03); however, this finding should be interpreted with caution, as cGVHD was analyzed as a fixed covariate rather than as a time-dependent variable. Therefore, this association likely reflects a survivor effect, since patients must survive long enough after transplantation to develop chronic GVHD, introducing a potential immortal time bias. The relatively limited sample size and number of events may have reduced the statistical power to detect modest differences between groups. Therefore, the findings should be interpreted with caution.

Overall, these results indicate that, although early Gram-negative infections remain clinically relevant and associated with higher infection-related mortality, their negative impact was mitigated by the structured infection management and targeted therapeutic strategies introduced under the BATMO protocol. Additionally, continuous inpatient monitoring during the first 30 days after transplant—through serial laboratory assessments and rapid clinical response to sepsis—allowed for timely antimicrobial intervention, likely offsetting the potential mortality burden associated with early BSI.

One of the primary goals of the BATMO program was to limit the emergence of antimicrobial resistance through rational antibiotic use [4, 5, 8–14]. The results strongly support the success of this objective. There was no significant increase in resistance among Gram-positive pathogens, despite the absence of fluoroquinolone prophylaxis. Even more strikingly, fluoroquinolone resistance among *E. coli* isolates decreased from 100% in the pre-BATMO cohort to 46% post-BATMO (*P* = .003), and overall resistance among all Gram-negative isolates fell from 75% to 36% (*P* = .006). These data collectively demonstrate a clear ecological benefit of discontinuing fluoroquinolone prophylaxis. Although a formal comparison with hospital-wide resistance trends was beyond the scope of this study, antimicrobial resistance among Gram-negative pathogens remains an increasing concern in hospitalized populations. In this context, the reduction in fluoroquinolone resistance observed in our transplant cohort after BATMO implementation may reflect the impact of a targeted antimicrobial stewardship strategy within this highly monitored patient population. Equally important, the use of carbapenems—last-resort antibiotics for multidrug-resistant infections—did not increase significantly in the post-BATMO era (43% vs 53%, *P* = .10). This suggests that the higher rate of BSI did not translate into an escalation of broad-spectrum antibiotic consumption, emphasizing the success of the protocol in preserving critical antimicrobial resources through a colonization-based, tailored empiric strategy.

Taken together, the five-year data confirm that the BATMO protocol is safe, effective, and sustainable over time. This conclusion becomes even more compelling when considering that the post-BATMO cohort was significantly enriched for high-risk transplants, particularly haploidentical grafts managed with PtCY, which historically carry higher NRM. Despite this shift, survival and NRM remained stable, underscoring the resilience of the stewardship approach. It successfully reduced antimicrobial resistance without compromising patient outcomes, preserved the efficacy of last-line antibiotics, and improved diagnostic clarity. Moreover, despite the discontinuation of fluoroquinolone prophylaxis and a more conservative antibiotic strategy, OS and NRM remained stable. In an era of escalating antimicrobial resistance, these findings provide compelling evidence in support of structured, evidence-based stewardship protocols tailored to transplant populations and to local epidemiology. These results confirm the long-term sustainability of the BATMO approach and support its broader adoption as a model for balancing infection control with antimicrobial preservation in immunocompromised patients.

## Supplementary Material

ofag511_Supplementary_Data

## Supplementary Data

Supplementary materials are available at *Open Forum Infectious Diseases* online. Consisting of data provided by the authors to benefit the reader, the posted materials are not copyedited and are the sole responsibility of the authors, so questions or comments should be addressed to the corresponding author.
