# Supplementary material for: Fluoroquinolone-Free Prophylaxis and Impact on Bacterial Infections, Resistance, and Survival in Allo-HSCT: 5-Year Follow-up of the BATMO Protocol (Best Anti-microbical Therapy Trapianto di Midollo Osseo)
Source: Open Forum Infect Dis. 2026 Aug 13;13(9):ofag511. doi: 10.1093/ofid/ofag511 (PMC13531467; doi:10.1093/ofid/ofag511)
Supplement: Supplementary file 1 [file ofag511_supplementary_data.docx]

**Supplementary Figure 1.** Distributions of Gram-positive (Supplemetary table 1A) and Gram-negative pathogens (Supplemetary table 1B) isolated in the first 30 days after SCT in the two *eras*

**A.**

**B.**

**TABLE 1S**

| **Variable** | **Comparison** | **HR** | **95% CI** | **P value** |
| --- | --- | --- | --- | --- |
| Age at transplant | Per 1-year increase | 1.00 | 0.99–1.02 | 0.600 |
| **Disease** | MDS vs AML | 1.04 | 0.57–1.90 | 0.888 |
|  | CML vs AML | 0.24 | 0.03–1.75 | 0.159 |
|  | CLL vs AML | 1.12 | 0.35–3.60 | 0.853 |
|  | ALL vs AML | 0.69 | 0.36–1.30 | 0.250 |
|  | Lymphoma vs AML | 0.94 | 0.53–1.68 | 0.837 |
|  | Other vs AML | 1.09 | 0.71–1.68 | 0.697 |
| **Donor type** | Related vs MUD | 0.83 | 0.55–1.23 | 0.347 |
|  | Haploidentical vs MUD | 0.83 | 0.50–1.38 | 0.473 |
|  | Cord blood vs MUD | 3.93 | 0.51–30.31 | 0.189 |
| **Transplant period** | Pre-BATMO vs BATMO | **1.43** | **0.97–2.10** | **0.068** |
| **PTCy prophylaxis** | Yes vs No | 1.53 | 0.89–2.62 | 0.124 |
| **HCT-CI** | Per 1-point increase | **1.16** | **1.07–1.26** | **<0.001** |
| **Stem cell source** | PBSC vs BM | 1.04 | 0.62–1.73 | 0.885 |
|  | Cord blood vs BM | NA* | NA | 0.994 |

Multivariable Cox proportional hazards model including transplant era, age, HCT‑CI, stem cell source, donor type, graft‑versus‑host disease prophylaxis (with PTCy included as a binary covariate), and disease category. Time was measured in months from transplantation.

**Table 2S**

| **Covariate** | **sHR** | **95% CI** | **p value** |
| --- | --- | --- | --- |
| **Era** |  |  |  |
| post‑BATMO vs pre‑BATMO | **0.666** | 0.366 – 1.211 | 0.180 |
| **Age (per year)** | 1.015 | 0.991 – 1.040 | 0.220 |
| **HCT‑CI (per point)** | **1.220** | **1.078 – 1.382** | **0.0017** |
| **Disease group** |  |  |  |
| AML/MDS | 0.674 | 0.271 – 1.674 | 0.400 |
| CML/CLL | 0.799 | 0.159 – 4.000 | 0.780 |
| Lymphoma | 1.470 | 0.509 – 4.244 | 0.480 |
| MM | 1.434 | 0.390 – 5.279 | 0.590 |
| Other | 1.334 | 0.455 – 3.912 | 0.600 |
| **PTCy** |  |  |  |
| yes vs no | 1.143 | 0.557 – 2.343 | 0.720 |

Multivariable Fine–Gray subdistribution hazards model evaluating factors associated with non‑relapse mortality (NRM), with relapse treated as a competing event. The model included transplant era (post‑BATMO vs pre‑BATMO), age at transplant, HCT‑CI score, disease category, stem cell source, and post‑transplant cyclophosphamide (PTCy; yes vs no). Results are reported as subdistribution hazard ratios (sHRs) with 95% confidence intervals (CI). Time was measured in months from transplantation.

**STROBE checklist for observational cohort studies**

STROBE checklist – BATMO study

STROBE Statement—Checklist of items that should be included in reports of cohort studies

Title and abstract

1a
Indicate the study’s design with a commonly used term in the title or abstract
Page 1–2

1b
Provide an informative and balanced summary of what was done and what was found
Page 2–3

Introduction

2
Explain the scientific background and rationale for the investigation being reported
Page 4–5

3
State specific objectives, including any prespecified hypotheses
Page 5

Methods

4
Present key elements of study design early in the paper
Page 7

5
Describe the setting, locations, and relevant dates, including recruitment, exposure, follow-up, and data collection
Page 7–8

6a
Eligibility criteria and methods of selection of participants
Page 7–8

6b
Matching criteria (if applicable)
Not applicable

7
Clearly define outcomes, exposures, predictors, and confounders
Page 8–9

8
Sources of data and methods of measurement
Page 7–9

9
Describe efforts to address potential sources of bias
Page 9–10

10
Explain how the study size was determined
Page 11

11
Explain how quantitative variables were handled in the analyses
Page 10

12a
Describe statistical methods used
Page 10–11

12b
Methods used to examine subgroups and interactions
Page 10–11

12c
Explain how missing data were addressed
Not applicable / no missing data reported

12d
Explain how loss to follow-up was addressed
Page 10

12e
Sensitivity analyses
Page 11

Results

13a
Report numbers of individuals at each stage of the study
Page 12

13b
Reasons for non-participation
Not applicable

13c
Consider use of a flow diagram
Not applicable

14a
Characteristics of study participants
Page 12–13

14b
Number of participants with missing data
Not applicable

14c
Summarise follow-up time
Page 11

15
Report numbers of outcome events over time
Page 13–15

16a
Provide estimates and confidence intervals
Page 14–16

16b
Report category boundaries if continuous variables categorized
Page 16

16c
Translate relative risk estimates into absolute risk if relevant
Not applicable

17
Other analyses (subgroup or sensitivity analyses)
Page 14–16

Discussion

18
Summarise key results with reference to study objectives
Page 17

19
Discuss study limitations and potential bias
Page 18–19

20
Provide cautious interpretation considering limitations and other studies
Page 17–20

21
Discuss generalisability of the results
Page 19–20

Other information

22
Source of funding and role of funders
Page 1
